# Genetic markers of bone and joint health and physical capability in older adults: the HALCyon programme

**DOI:** 10.1016/j.bone.2012.10.004

**Published:** 2013-01

**Authors:** Tamuno Alfred, Yoav Ben-Shlomo, Rachel Cooper, Rebecca Hardy, Cyrus Cooper, Ian J. Deary, David Gunnell, Sarah E. Harris, Meena Kumari, Richard M. Martin, Avan Aihie Sayer, John M. Starr, Diana Kuh, Ian N.M. Day, the HALCyon study team

**Affiliations:** a School of Social and Community Medicine, University of Bristol, Bristol BS8 2PS, UK; b MRC Unit for Lifelong Health and Ageing and Division of Population Health, University College London, London WC1B 5JU, UK; c MRC Lifecourse Epidemiology Unit, University of Southampton, Southampton SO16 6YD, UK; d Institute of Musculoskeletal Sciences, University of Oxford, Oxford OX3 7LD, UK; e Centre for Cognitive Ageing and Cognitive Epidemiology, University of Edinburgh, Edinburgh EH8 9JZ, UK; f Department of Psychology, University of Edinburgh, Edinburgh EH8 9JZ, UK; g Medical Genetics Section, University of Edinburgh, Edinburgh EH4 2XU, UK; h Department of Epidemiology and Public Health, University College London, London WC1E 6BT, UK; i MRC Centre for Causal Analyses in Translational Epidemiology, School of Social and Community Medicine, University of Bristol, Bristol BS8 2BN, UK; j Academic Geriatric Medicine, University of Southampton, Southampton General Hospital, Southampton SO16 6YD, UK; k Alzheimer Scotland Dementia Research Centre, University of Edinburgh, 7 George Square, Edinburgh EH8 9JZ, UK

**Keywords:** BMD, bone mineral density, OA, osteoarthritis, BMI, body mass index, SNP, single nucleotide polymorphism, CaPS, Caerphilly Prospective Study, ELSA, English Longitudinal Study of Ageing, HAS, Hertfordshire Ageing Study, HCS, Hertfordshire Cohort Study, LBC1921, The Lothian Birth Cohort 1921, NSHD, National Survey of Health and Development, HWE, Hardy–Weinberg equilibrium, WHR, waist–hip ratio, GWAS, genome-wide association studies, Aging, Grip strength, Calcium, Bone mineral density, Osteoarthritis

## Abstract

**Background:**

Good bone and joint health is essential for the physical tasks of daily living and poorer indicators of physical capability in older adults have been associated with increased mortality rates. Genetic variants of indicators of bone and joint health may be associated with measures of physical capability.

**Methods:**

As part of the Healthy Ageing across the Life Course (HALCyon) programme, men and women aged between 52 and 90 + years from six UK cohorts were genotyped for a polymorphism associated with serum calcium (rs1801725, *CASR*), two polymorphisms associated with bone mineral density (BMD) (rs2941740, *ESR1* and rs9594759, *RANKL*) and one associated with osteoarthritis risk rs3815148 (*COG5*). Meta-analysis was used to pool within-study effects of the associations between each of the polymorphisms and measures of physical capability: grip strength, timed walk or get up and go, chair rises and standing balance.

**Results:**

Few important associations were observed among the several tests. We found that carriers of the serum calcium-raising allele had poorer grip strength compared with non-carriers (pooled *p* = 0.05, *n* = 11,239) after adjusting for age and sex. Inconsistent results were observed for the two variants associated with BMD and we found no evidence for an association between rs3815148 (*COG5*) and any of the physical capability measures.

**Conclusion:**

Our findings suggest elevated serum calcium levels may lead to lower grip strength, though this requires further replication. Our results do not provide evidence for a substantial influence of these variants in *ESR1*, *RANKL* and *COG5* on physical capability in older adults.

## Highlights

► We examined associations between bone-related genotypes and physical capability. ► We conducted a meta-analysis on 12,836 middle-age adults. ► We found CASR may be associated with grip strength. ► No substantial support for specific bone mineral density variants and physical capability.

## Introduction

Bones have several important functions, including, providing support, permitting movement and storing minerals [1]. The indicators of poorer bone health observed in later life have been associated with adverse outcomes such as increased morbidity [2], mortality [2,3] and low grip strength [4]. Objective measures of physical capability, the capacity to undertake the physical tasks of daily living, including grip strength, have themselves been associated with morbidity [5] and mortality [6] rates. Therefore, understanding the contributors to bone health may be informative to the maintenance of good levels of physical capability in later life.

One of the most prominent minerals in bone is calcium [7] and it is vital for bone health. Dietary calcium intake that reduces its deficiency may increase or maintain bone mass [8,9] and evidence suggests that women in the lowest quintile of calcium intake have the highest risk of first fracture and osteoporosis [10]. Much of the 1% of calcium that is not stored in the bones and teeth of adult humans is found in the bloodstream and whilst serum calcium may not be an indicator of calcium intake [11], its tight regulation is a driver of bone calcium resorption [12], suggesting it may also be important to bone-related outcomes. Lower levels of serum calcium have been associated with increased risk of vertebral fractures [13], though there is so far little evidence for associations with physical capability [14,15]. In addition to factors such as immobility [16] and inactivity [17] being associated with higher serum calcium in the elderly, there is also a genetic component, with an estimated 33% heritability [18], and genome-wide association studies (GWAS) have found that the T allele of SNP rs1801725 (A986S) of the calcium-sensing receptor gene (*CASR*) is associated with increased serum calcium [19,20].

Bone mineral density (BMD) declines from mid-life, particularly sharply in women after menopause [21]. BMD explains around 60% of the variability of bone compression strength [22], is used in the diagnosis of osteoporosis [23] and is a predictor of fracture risk [24]. Common sites for BMD measurement are the hip and lumbar spine, with moderate correlation between the two [25]. Lower levels of BMD at these sites have been associated with poorer measures of physical capability, including grip strength and walking speed [4,26]. BMD and rates of bone loss in later life may be modified by exercise programs [27], cigarette smoking [28] and fat mass [29,30] in addition to having a substantial genetic component, with heritability estimates of 77% and 89% for hip and lumbar spine, respectively [31]. From GWAS, the G allele of rs2941740, near *ESR1*, has been associated with increased hip and lumbar spine BMD [32] and the C allele of rs9594759 near *TNFSF11* (aka *RANKL*) has been associated with increased lumbar spine BMD [32–34], along with some evidence for an association with hip BMD [34].

Osteoarthritis (OA) is the most common joint disease and in addition to age and obesity [35], its risk may also be influenced by bone quality [36]. OA at different sites has been associated with poorer physical capability, such as slower 6 m walking speeds for hip OA [37] and lower grip strength in individuals with hand OA [38]. Genetic variants contributing to the estimated at least 40% heritability for hand and knee OA [39] have been identified from GWAS, with the C allele of SNP rs3815148 in *COG5* associated with increased risk of knee and/or hand OA [40].

We therefore hypothesised that SNPs associated with markers of bone and joint health would be associated with levels of physical capability. To investigate this we analysed data from 12,836 participants aged between 52 and 90 + years as part of the HALCyon (Healthy Ageing across the Life Course; www.halcyon.ac.uk) collaborative research programme in what we believe to be the largest investigation into associations between these polymorphisms and physical capability.

## Methods

### Study populations

The Medical Research Council National Survey of Health and Development (NSHD) comprises participants sampled from all births in a week in March 1946 and followed up since. In 1999, at age 53 years, men and women were visited by a research nurse and consent for DNA extraction was given by approximately 2900 members of the cohort. Details of the data collected and the several phases of the study are available on the cohort's website (www.nshd.mrc.ac.uk) and elsewhere [41].

The English Longitudinal Study of Ageing (ELSA) comprises men and women aged 50 years and over who originally participated in the Health Survey for England in 1998, 1999 or 2001. Fieldwork began in 2002–03 (Phase I) with two-yearly follow-ups and in 2004–05 (Phase II) blood samples were provided by 6231 participants. Details of the cohort have been published [42].

The Hertfordshire Cohort Study (HCS) consists of 2997 participants born 1931–39 and registered with a General Practitioner in East, North or West Hertfordshire who attended a clinic in 1994–2004 (Phase I). A second assessment took place in 2004–05 for participants in East Hertfordshire (Phase II). Further details of study design, data collected and summaries of participant characteristics have been published [43] and are available on its website (www.mrc.soton.ac.uk/herts).

The Boyd Orr cohort is a historical cohort study based on children surveyed in 1937–39 in English and Scottish districts. Participants were followed-up for vital status via the NHS Medical Information Research Service (MIRS) since 1948, with questionnaire administration to survivors in 1997–98 (Phase II) and a research clinic visit in 2002–03 (Phase III), during which DNA was extracted from 728 adults, of which 385 had at least one physical capability measure and one relevant genotype called for this analysis. Details of the study design and the data collected have been described on its website (www.epi.bris.ac.uk/boydorr) and elsewhere [44].

The Caerphilly Prospective Study (CaPS) recruited 2512 men aged between 45 and 59 years in 1979–83 from the town of Caerphilly, South Wales, and its surrounding villages. Blood samples were collected at baseline and at each of the four follow-ups (Phase II: 1984–88, Phase III: 1989–93, Phase IV: 1993–97 and Phase V: 2002–04.) Further details are available on the cohort's website (www.epi.bris.ac.uk/caerphilly/caerphillyprospectivestudy.htm).

The Lothian Birth Cohort 1921 (LBC1921) participants were all born in 1921 and most completed a cognitive ability assessment at age 11 years. In 1999–2001 (Wave I), at approximately 79 years old, 550 participants living in and around Edinburgh, underwent a series of cognitive and physical tests. Details of the recruitment into the study are available on its website (www.lothianbirthcohort.ed.ac.uk) and have been published previously [45,46].

### Genotyping and quality control

Genotyping for SNPs rs1801725 (A986S, *CASR*), rs2941740 (*ESR1*), rs9594759 (*RANKL*) and rs3815148 (*COG5*) was carried out by KBioscience (www.kbioscience.co.uk) for the majority of studies. Genotype information for rs1801725 in NSHD came from the Illumina Metabochip (www.illumina.com). Genotype information for rs1801725 and rs2941740 (using proxy rs3020331) came from the Illumina Human 610-Quadv1 Chip in LBC1921 [47]. Data quality was reviewed by assessing clustering quality (using KBioscience software SNPviewer on their data), call rates and deviation from Hardy–Weinberg equilibrium (HWE).

### Phenotypes

#### Anthropometry

Measurements were conducted either at clinics, during a clinical interview in the home, or from self-report. Body mass index (BMI kg/m^2^) was calculated as weight (kg) divided by height (m) squared. Waist–hip ratio (WHR) was defined as waist circumference (cm) divided by hip circumference (cm) and was measured in NSHD, ELSA, HCS, HAS, Boyd Orr and CaPS.

#### Physical capability

Grip strength was measured in NSHD, ELSA, HCS and LBC1921 using electronic or hydraulic dynamometers, with the best measure used in the analysis where more than one trial was conducted. Standing balance tests were conducted in the studies, with participants’ eyes open: flamingo [48] (stopped at 30 s) in NSHD, HCS, Boyd Orr and CaPS, and side-by-side, semi-tandem and full tandem [49] in ELSA. Ability to balance was defined in this analysis as the ability to complete at least 5 s. The timed get up and go test [50] was carried out in HCS, Boyd Orr and CaPS and required participants to get up from a chair, walk 3 m, turn, walk back, turn and sit down. Timed walks over 2.44 m (8 ft) and 6 m were carried out in ELSA and LBC1921 respectively. Speeds were calculated for timed walks and get up and go, with the fastest speeds used in the analysis where more than one trial was conducted. Timed chair rises [51] involved asking participants to rise from a chair and sit back down 5 times in ELSA and HCS and 10 times in NSHD; the reciprocal of time taken in seconds × 100 [52] was used in the analysis. Further details of these measurements in these cohorts are presented elsewhere [53].

#### Demographic information

Demographic information was derived from self-reports. Where information on ethnicity was collected, participants of non-European ancestry were excluded from analyses to avoid confounding from population stratification [54]. Levels of physical activity were derived from questionnaires in NSHD, ELSA, Boyd Orr, CaPS and LBC1921. Individuals were categorised as ‘physically active’ in this analysis if they engaged, at least once a month, in at least moderate sport or activities in NSHD, Boyd Orr, CaPS and LBC1921 or vigorous sport or activities in ELSA. Participants’ alcohol consumption was dichotomised here into ‘at least weekly’ and less often in all studies, except NSHD, where ‘more often than special occasions’ and less often were used. Data on current smoking status and socio-economic position were also used.

### Statistical methods

Within studies, linear and logistic regression analyses were conducted on the continuous and dichotomous traits within the cohorts respectively, adjusting for sex in all studies except CaPS, and age in all studies except NSHD and LBC1921. Adjustments for anthropometric and other demographic variables were made where appropriate. Due to the low frequency of individuals homozygous for the T allele of rs1801725 (*n* = 207, 1.7%) and the C allele of rs3815148 (*n* = 637, 5.1%), dominant models were used for these polymorphisms in order to avoid the presentation of tables containing cells with very low frequencies in particular cohorts. Additive models were used for rs2941740 and rs9594759 with genotypes coded as 0, 1 and 2 for the number of minor alleles. Likelihood ratio tests were used to compare the fit of the additive models compared with the full genotype model. For continuous traits, the normality of the standardised residuals was inspected with distributional diagnostic plots. For the harmonisation of continuous traits that were used to obtain pooled estimates of the genotypic effects, z-score units were calculated in each study by subtracting the study mean and dividing by its standard deviation. The overall mean for z-scores is 0 and standard deviation 1. Two-step [55] meta-analyses using a random-effects model were performed to obtain pooled genotypic effects. The I^2^ measure was used to quantify heterogeneity [56]. Finally, the calculation of z-scores, for the continuous traits, and the main analyses were repeated in males and females separately. Reporting of the analyses met the appropriate items of recommended checklists [57,58]. A two-tailed significance level of *p* < 0.05 was used as evidence of statistical significance. Statistical analysis was performed in Stata 11.2 (StataCorp LP).

## Results

### Cohort summaries and genotyping quality

A total of 12,836 adults aged between 52 and 90 + years had relevant genotypic and phenotypic data available (Table 1). Summaries of measures of body size and demographic characteristics are presented in Table S1. The call rates were high, exceeding 93% across all studies for the four polymorphisms. The HWE condition was met in all studies for all polymorphisms (*p* > 0.08), except for rs9594759 (*RANKL*) in NSHD (*p* = 0.009) and CaPS (*p* = 0.04).

### Associations between genotypes and phenotypes

Associations between the genotypes and anthropometric and demographic variables are presented in Tables S2–S4, showing no evidence for genotypic effects on any of the considered potential confounders for physical capability in the pooled analyses, except for alcohol consumption for rs9594759 (*RANKL*), with the C allele less common among frequent drinkers (*p* = 0.004, Fig. S1).

**Fig. 1:**
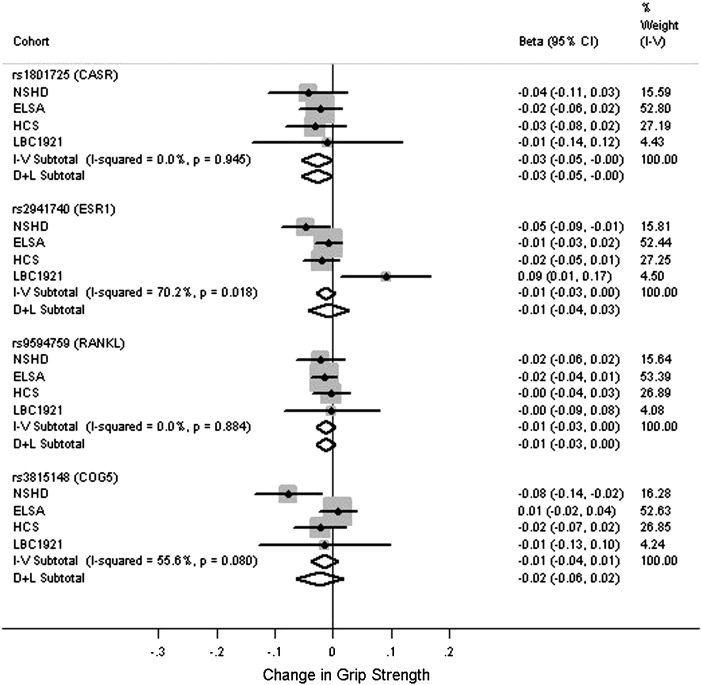
Meta-analyses for the associations between genotypes and grip strength. Adjusted for age and sex. Coefficients based on z-scores. Models used: rs1801725 – (G/T + T/T) vs. G/G; rs2941740 – per minor (G) allele; rs9594759 – per minor (C) allele; rs3815148 – (A/C + C/C) vs. A/A.

**Fig. 2:**
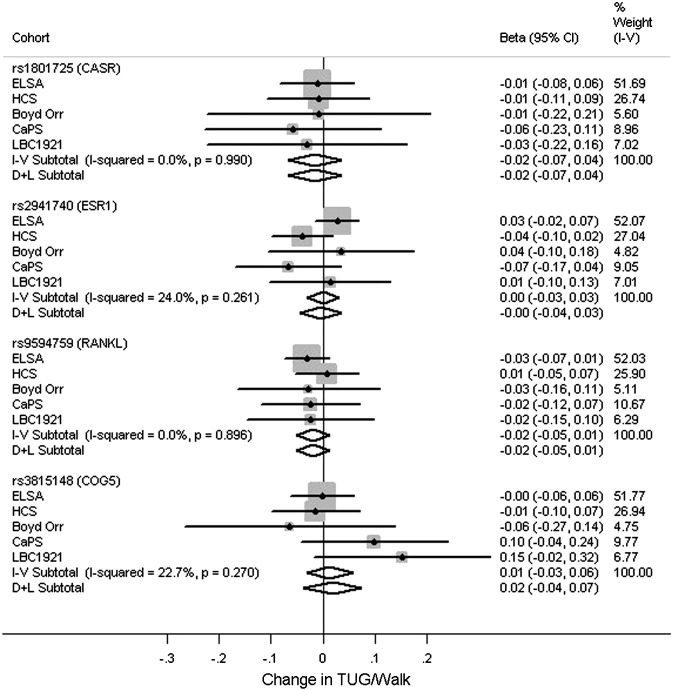
Meta-analyses for the associations between genotypes and timed get up and go/walk speed. Adjusted for age and sex. Coefficients based on z-scores. Models used: rs1801725 – (G/T + T/T) vs. G/G; rs2941740 – per minor (G) allele; rs9594759 – per minor (C) allele; rs3815148 – (A/C + C/C) vs. A/A.

**Fig. 3:**
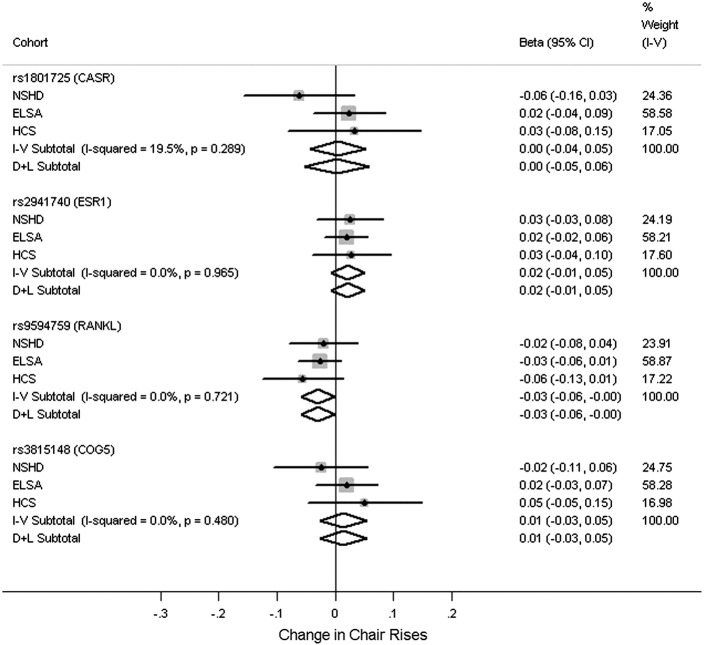
Meta-analyses for the associations between genotypes and chair rises. Adjusted for age and sex. Coefficients based on z-scores. Timed chair rises on reciprocal of time taken in seconds × 100. Models used: rs1801725 – (G/T + T/T) vs. G/G; rs2941740 – per minor (G) allele; rs9594759 – per minor (C) allele; rs3815148 – (A/C + C/C) vs. A/A.

**Fig. 4:**
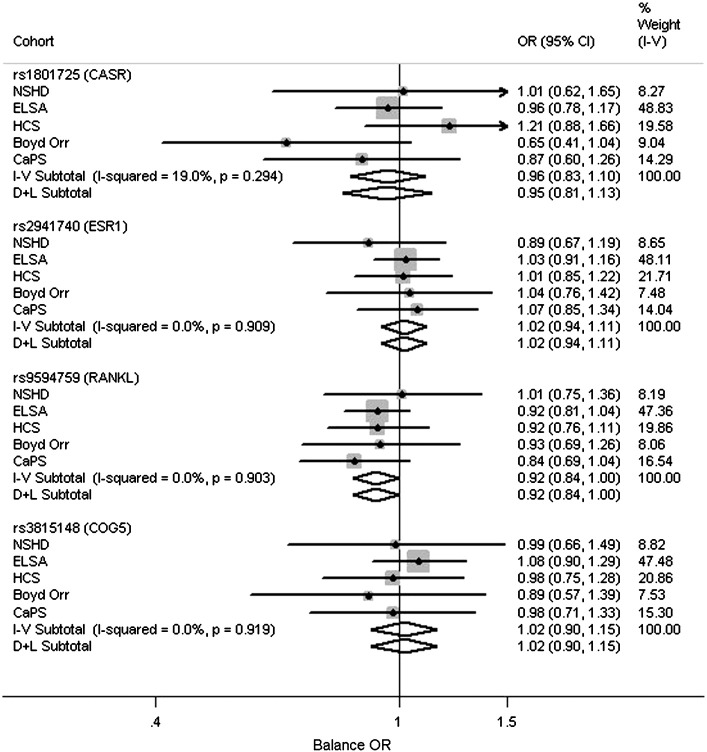
Meta-analyses for the associations between genotypes and ability to balance for at least 5 s. Adjusted for age and sex. Models used: rs1801725 – (G/T + T/T) vs. G/G; rs2941740 – per minor (G) allele; rs9594759 – per minor (C) allele; rs3815148 – (A/C + C/C) vs. A/A.

Figs. 1–4 and Tables S5–S8 show the associations between the polymorphisms and measures of physical capability adjusted for age and sex. From the pooled analyses there was some evidence for an association between the T allele of rs1801725 (*CASR*) and poorer grip strength (*p* = 0.05). There were no associations observed between any of the genotypes and timed get up and go/walk speed (*p* > 0.2). There was evidence for an association between the C allele of rs9594759 and slower chair rise times (*p* = 0.04). There was evidence for an association between the C allele of rs9594759 and poorer standing balance (*p* = 0.04), although this effect was only seen in females with some evidence for a sex difference (*p* = 0.05 for heterogeneity between males and females, Fig. S2). There was evidence for heterogeneity between males and females for the association between rs3815148 (*COG5*) and standing balance, (*p* = 0.012, Fig. S3) with the observed effects in opposite directions. No other genotypic associations with physical capability measures or evidence for sex differences were observed.

Additional adjustment for alcohol consumption for the genotypic effects of rs9594759 did not substantially affect its associations with chair rises (pooled beta for z-score = − 0.031, 95% CI: − 0.060 to − 0.002, *p* = 0.04, *n* = 8184) and standing balance in females (pooled OR = 0.85, 95% CI: 0.75–0.96, *p* = 0.01, data not shown).

In only a relatively small number of tests did the full genotype model represent a significantly better fit than the per allele model: rs9594759 for weight and BMI in Boyd Orr, smoking status and timed walk in LBC1921; rs2941740 for smoking status in ELSA, socio-economic position in NSHD and balance in CaPS.

## Discussion

In this large, multi-cohort study of older adults we investigated associations between robust genetic markers of serum calcium, bone mineral density and osteoarthritis risk and measures of physical capability in six UK cohorts of 12,836 adults aged between 52 and 90 + years. We found marginal evidence for an association between rs1801725 (*CASR*) and grip strength, with carriers of the allele associated with raised serum calcium levels, identified from GWAS [19,20], having lower grip strength. However, the effect size was small at − 0.03 z-score units for carriers of the T allele, adjusting for age and sex, representing 0.33 kg assuming a standard deviation of 11. We also found some evidence for the association of the BMD-raising allele (C) of rs9594759 (*RANKL*) [32–34] with slower chair rise times and poorer standing balance. This direction was unexpected; however, the interpretation of these results should be treated with caution as the HWE condition was not met for rs9594759 (*RANKL*) in NSHD and CaPS, and whilst exclusion of the studies is not recommended [59], both studies contributed to the meta-analysis for standing balance and NSHD also contributed to that for chair rises. There were no observed associations with the physical capability measures for the BMD-raising allele of rs2941740 (*ESR1*). To the best of our knowledge, this is the first report of an investigation between a polymorphism in the *COG5* region and physical capability and we found no evidence that carriers of the risk allele for osteoarthritis of rs3815148 had poorer physical capability measures in the combined analyses of men and women. Due to the sex differences in bone loss rate in later life [21], we additionally investigated genotypic effects separately in men and women; we found borderline evidence for a difference in the effects of rs9594759 (*RANKL*) on standing balance by sex, with the effects only observed in women, and opposing directions of effect for rs3815148 (*COG5*) on standing balance, though we found no evidence for other differences.

Genetic variants are generally not associated with typical confounders in observational epidemiology and, being fixed from conception, may be informative about the direction of causality [60]. We found no evidence of association between rs1801725 (*CASR*) and measures of anthropometry, physical activity levels or other demographic indicators. Additionally, previous investigations of *CASR* polymorphisms have found evidence against associations with many traits including, vitamin D levels [61], osteoarthritis [19], osteoporosis [19] or hip BMD [19,20], as well as no[19] or only modest [20] associations with lumbar spine BMD; however, there is some evidence that their effects on BMD may be modified by birth-weight [62]. Although a previous smaller study of 1252 females aged between 70 and 85 years found no associations between the SNP and either grip strength or timed up and go [17], our findings based on a larger number of individuals (*n* = 11,239) suggest that our observed association between rs1801725 and grip strength may indicate a causal role of raised serum calcium levels on poorer grip strength. The association observed with grip strength but not with the three other physical capability phenotypes may be indicative of greater power due to the larger number of participants with available data with this trait. The inconsistent findings for the direction of effects for the BMD-raising alleles of the two SNPs considered and the associations observed for rs9594759 (*RANKL*) suggest further investigations are warranted in order to provide additional evidence for or against the causal role of BMD on physical capability. Previous smaller studies of older females (*n* = 421 [63] and 331 [64]) found no association between measures of physical performance, including grip strength, and SNP rs2234693, a variant in low LD (*r*^2^ = 0.04) with rs2941740 (*ESR1*).

Our investigation was limited by the fact that we did not validate the genotypic effects of the SNPs on serum calcium, BMD or osteoarthritis in these studies. However, all of the SNPs chosen were robustly associated with their respective measures from large GWAS of individuals of European ancestry. The use of younger populations may help to elucidate whether associations are present at earlier stages of the life course. Our survey of the literature for SNP selection for this study was conducted prior to the emergence of evidence for further genotypic associations with BMD [65] and OA [66] and, therefore, additional insight could be gained from other large investigations using new loci for associations with these traits.

It is clear that adequate calcium intake is essential for bone health [8,9] as well as having other benefits, including possibly protecting against obesity [67]. However, calcium intake above the level required by bones is likely to be excreted through the urine [7] and there is even evidence that higher levels of calcium intake (greater than around 1100 mg) may increase the risk of hip fractures [10]. Higher levels of serum calcium may have other adverse consequences including increased cardiovascular and mortality risk [68]. Hypocalcaemia is also associated with muscle weakness and fatigue and a small study of patients with primary hyperparathyroidism found the post-surgical reduction in serum calcium was correlated with improved strength [69]. Our use of a genetic variant of serum calcium provides additional insight into the effects of long-term raised serum calcium levels on measures of physical capability. As a result, greatly exceeding the UK recommendation of 700 mg calcium per day for adults [70] is not advised, and a preference for food sources over pharmacological supplements may lead to smaller effects on serum levels [68,71]. Whilst further studies are needed to infer causality between BMD and physical capability, attention should still be paid to the modifiable factors of bone mass, such as exercise programs [27], that would be beneficial to osteoporosis risk [23,24] as well as to the maintenance of good physical capability.

### Conclusion

The results of this large multi-cohort study of older adults suggest elevated serum calcium levels may lead to lower grip strength but provide no evidence for its effect on other measures of physical capability. Genetic markers of BMD and osteoarthritis risk provided null or inconsistent associations with measures of physical capability.

## Figures and Tables

**Table 1:** Summary of sex, age and minor allele frequencies by cohort.

				Cohort			
Characteristic	NSHD	ELSA	HCS	Boyd Orr	CaPS	LBC1921	Total
Number of participants	2646	5612	2892	385	788	513	12836
Male, %	50	46	53	45	100	41	51
Age^a^ in years, median (range)	53	65 (52–90 +)	66 (59–73)	70 (64–82)	72 (65–83)	79 (77–80)	64 (52–90 +)
Minor allele frequencies							
*CASR*- rs1801725,T	0.13	0.13	0.13	0.14	0.11	0.14	0.13
*ESR1*- rs2941740, G	0.42	0.42	0.44	0.38	0.40	0.48	0.43
*RANKL*- rs9594759, C	0.44	0.45	0.45	0.44	0.45	0.43	0.45
*COG5*- rs3815148, C	0.23	0.23	0.23	0.19	0.22	0.25	0.23

Numbers of participants represent those with available data for at least one physical capability phenotype and at least one genotype. ^a^Age at phase from which the majority of variables are taken, i.e. Boyd Orr: III; CaPS: V; ELSA: II; HCS: I; LBC1921: I; NSHD: 1999 Collection; CaPS: Caerphilly Prospective Study; ELSA: English Longitudinal Study of Ageing; HCS: Hertfordshire Cohort Study; LBC1921: Lothian Birth Cohort 1921; NSHD: National Survey of Health and Development.
