# Supplementary material for: Genetic markers of bone and joint health and physical capability in older adults: the HALCyon programme
Source: Bone. 2013 Jan;52(1):278–85. doi: 10.1016/j.bone.2012.10.004 (PMC3526776; doi:10.1016/j.bone.2012.10.004)
Supplement: Supplementary file 1 — Supplementary materials. [file mmc1.doc]

Table S1Summary of Body Size and Demographic Characteristics by Cohort

|  |  |  | Cohort |  |  |  |
| --- | --- | --- | --- | --- | --- | --- |
| Characteristic | NSHD | ELSA | HCS | Boyd Orr | CaPS | LBC1921 |
| Height in cm, mean (sd) | 168.2 (8.9) | 165.7 (9.5) | 167.9 (9.1) | 164.2 (9.3) | 170.2 (6.2) | 163.2 (9.3) |
| Weight in kg, mean (sd) | 77.5 (14.7) | 76.4 (15.3) | 77.2 (14.2) | 74.4 (14.0) | 80.9 (12.7) | 69.9 (12.7) |
| BMI in kg/m^2^, mean (sd) | 27.3 (4.6) | 27.8 (4.7) | 27.4 (4.4) | 27.5 (4.4) | 27.9 (4.0) | 26.2 (4.1) |
| WHR, mean (sd) | 0.87 (0.09) | 0.89 (0.08) | 0.91 (0.08) | 0.91 (0.09) | 0.93 (0.06) | - |
| Physical activity, active % | 51 | 41 | - | 82 | 55 | 51 |
| Alcohol^a^, at least weekly % | 73 | 63 | 70 | 48 | 64 | 76 |
| Smoking status, current/ex % | 71 | 62 | 54 | - | 73 | 56 |
| SEP, professional/managerial % | 44 | 36 | 23 | 42 | 28 | 58 |

1. NSHD: more frequently than on special occasions. SEP: socio-economic position. NB CaPS only contains male participants

**Table S2 Summary of Pooled Associations between rs1801725 (*CASR*)** and Potential Confounders for Physical Capability

| Potential Confounder | Beta (95% CI) | p | I^2^ %; Het p | N |
| --- | --- | --- | --- | --- |
| Height, cm | 0.02 (-0.01- 0.04) | 0.28 | 0.0; 0.81 | 12342 |
| Weight, kg | 0.01 (-0.03- 0.05) | 0.57 | 1.2; 0.41 | 12352 |
| BMI, kg/m^2^ | 0.00 (-0.04- 0.05) | 0.91 | 12.4; 0.34 | 12256 |
| WHR | 0.03 (-0.01- 0.06) | 0.09 | 0.0; 0.95 | 11851 |
|  |  | | | |
|  | OR (95% CI) | p | I^2^ %; Het p | n/N |
| Physical activity, active vs. not | 1.01 (0.88- 1.16) | 0.93 | 30.4; 0.22 | 4486/9562 |
| Alcohol, weekly vs. less frequent | 0.94 (0.85- 1.03) | 0.16 | 0.0; 0.56 | 7972/11896 |
| Smoking status, current/ex vs. never | 1.08 (0.97- 1.21) | 0.18 | 25.0; 0.26 | 7478/12021 |
| SEP, professional/managerial vs. other | 0.99 (0.91- 1.08) | 0.85 | 0.0; 0.98 | 4328/12218 |

SEP: socio-economic position. Coefficients for rs1801725: G/T+T/T vs. G/G. Models for height, weight, BMI and WHR based on z-scores. All coefficients adjusted for age and sex.

**Table S3 Summary of Pooled Associations between rs2941740 (*ESR1*) and rs9594759 (*RANKL*)** and Potential Confounders for Physical Capability

| Potential | rs2941740 (*ESR1*) | | | | rs9594759 (*RANKL*) | | | |
| --- | --- | --- | --- | --- | --- | --- | --- | --- |
| Confounder | Beta (95% CI) | p | I^2^ %; Het p | N | Beta (95% CI) | p | I^2^ %; Het p | N |
| Height, cm | 0.00 (-0.02- 0.02) | 0.94 | 0.0; 0.99 | 12418 | -0.01 (-0.02- 0.01) | 0.61 | 0.0; 0.54 | 12249 |
| Weight, kg | -0.01 (-0.04- 0.02) | 0.42 | 9.9; 0.35 | 12430 | 0.01 (-0.02- 0.03) | 0.70 | 0.0; 0.82 | 12261 |
| BMI, kg/m^2^ | -0.01 (-0.05- 0.02) | 0.38 | 24.1; 0.25 | 12332 | 0.01 (-0.02- 0.03) | 0.50 | 0.0; 0.55 | 12165 |
| WHR | 0.00 (-0.02- 0.02) | 0.87 | 0.0; 0.51 | 11929 | 0.01 (-0.02- 0.03) | 0.63 | 38.0; 0.17 | 11772 |
|  |  |  |  |  |  |  |  |  |
|  | OR (95% CI) | p | I^2^ %; Het p | n/N | OR (95% CI) | p | I^2^ %; Het p | n/N |
| Physical activity, active vs. not | 0.98 (0.93- 1.04) | 0.59 | 0.0; 0.65 | 4506/9622 | 0.99 (0.93- 1.05) | 0.77 | 0.0; 0.43 | 4454/9483 |
| Alcohol, weekly vs. less frequent | 1.04 (0.98- 1.10) | 0.21 | 0.0; 0.61 | 8007/11973 | 0.92 (0.87- 0.97) | 0.0036 | 0.0; 0.71 | 7938/11830 |
| Smoking status, current/ex vs. never | 0.99 (0.94- 1.05) | 0.78 | 0.0; 0.50 | 7507/12111 | 0.96 (0.88- 1.04) | 0.28 | 44.4; 0.13 | 7427/11940 |
| SEP, professional/managerial vs. other | 1.00 (0.95- 1.06) | 0.95 | 0.0; 0.45 | 4346/12295 | 1.04 (0.96- 1.13) | 0.30 | 41.4; 0.13 | 4293/12130 |

SEP: socio-economic position. Coefficients for rs2941740: per G allele; rs9594759: per C allele. Models for height, weight, BMI and WHR based on z-scores. All coefficients adjusted for age and sex.

**Table S4 Summary of Pooled Associations between rs3815148 (*COG5*)** and Potential Confounders for Physical Capability

| Potential Confounder | Beta (95% CI) | p | I^2^ %; Het p | N |
| --- | --- | --- | --- | --- |
| Height, cm | -0.01 (-0.04- 0.01) | 0.36 | 0.0; 0.99 | 12422 |
| Weight, kg | -0.03 (-0.06- 0.01) | 0.13 | 0.0; 0.58 | 12434 |
| BMI, kg/m^2^ | -0.02 (-0.06- 0.01) | 0.23 | 0.0; 0.50 | 12336 |
| WHR | 0.01 (-0.02- 0.03) | 0.62 | 0.0; 0.64 | 11934 |
|  |  | | | |
|  | OR (95% CI) | p | I^2^ %; Het p | n/N |
| Physical activity, active vs. not | 1.04 (0.96- 1.14) | 0.33 | 0.0; 0.73 | 4519/9633 |
| Alcohol, weekly vs. less frequent | 0.95 (0.88- 1.03) | 0.25 | 0.0; 0.94 | 8010/11969 |
| Smoking status, current/ex vs. never | 1.02 (0.92- 1.12) | 0.76 | 29.8; 0.22 | 7504/12102 |
| SEP, professional/managerial vs. other | 0.98 (0.89- 1.06) | 0.57 | 12.3; 0.34 | 4350/12295 |

SEP: socio-economic position. Coefficients for rs3815148: A/A vs. C/C+A/C. Models for height, weight, BMI and WHR based on z-scores. All coefficients adjusted for age and sex.

**Table S5 Physical Capability by rs1801725 (*CASR*)** Genotype and Cohort

| Variable | Cohort | G/G | G/T+T/T | Total | b (95% CI)^a^ | p-value | Heterogeneity |
| --- | --- | --- | --- | --- | --- | --- | --- |
|  |  | mean (sd) [n] | mean (sd) [n] | mean (sd) [n] |  |  | I^2^%; p-value |
|  | NSHD | 37.9 (14.3) [1796] | 37.6 (14.8) [572] | 37.8 (14.4) [2368] | -0.04 (-0.11- 0.03) | 0.22 |  |
|  | ELSA | 32.2 (11.6) [4227] | 31.9 (11.5) [1300] | 32.1 (11.6) [5527] | -0.02 (-0.06- 0.02) | 0.25 |  |
| Grip strength, kg | HCS | 35.9 (11.0) [2158] | 35.3 (11.1) [673] | 35.8 (11.0) [2831] | -0.03 (-0.08- 0.02) | 0.24 |  |
|  | LBC1921 | 26.5 (9.0) [381] | 26.5 (9.5) [132] | 26.5 (9.1) [513] | -0.01 (-0.14- 0.12) | 0.87 |  |
|  | **Pooled** | **[8562]** | **[2677]** | **[11239]** | **-0.027 (-0.054- -0.000)** | **0.0498** | **0.0; 0.95** |
| Timed 2.44m walk, m/s | ELSA | 0.93 (0.30) [2701] | 0.93 (0.30) [824] | 0.93 (0.30) [3525] | -0.01 (-0.08- 0.06) | 0.76 |  |
| Timed 3m get up and go, m/s | HCS | 0.57 (0.10) [1603] | 0.57 (0.09) [509] | 0.57 (0.10) [2112] | -0.01 (-0.11- 0.09) | 0.86 |  |
| Timed 3m get up and go, m/s | Boyd Orr | 0.67 (0.16) [278] | 0.67 (0.13) [106] | 0.67 (0.16) [384] | -0.01 (-0.22- 0.21) | 0.94 |  |
| Timed 3m get up and go, m/s | CaPS | 0.60 (0.13) [594] | 0.59 (0.14) [154] | 0.60 (0.13) [748] | -0.06 (-0.23- 0.11) | 0.51 |  |
| Timed 6m walk, m/s | LBC1921 | 1.41 (0.39) [379] | 1.40 (0.33) [131] | 1.40 (0.37) [510] | -0.03 (-0.22- 0.16) | 0.76 |  |
|  | **Pooled** | **[5555]** | **[1724]** | **[7279]** | **-0.016 (-0.067- 0.035)** | **0.55** | **0.0; 0.99** |
| Timed chair rises^b^-10 rises | NSHD | 5.22 (1.68) [1749] | 5.12 (1.63) [547] | 5.20 (1.67) [2296] | -0.06 (-0.16- 0.03) | 0.20 |  |
| Timed chair rises^b^-5 rises | ELSA | 9.62 (3.16) [3683] | 9.69 (3.17) [1125] | 9.63 (3.16) [4808] | 0.02 (-0.04- 0.09) | 0.45 |  |
| Timed chair rises^b^-5 rises | HCS | 6.24 (1.47) [1146] | 6.27 (1.51) [354] | 6.24 (1.48) [1500] | 0.03 (-0.08- 0.15) | 0.57 |  |
|  | **Pooled** | **[6578]** | **[2026]** | **[8604]** | **0.002 (-0.052- 0.057)** | **0.93** | **20; 0.29** |
|  |  |  |  |  |  |  |  |
| Variable | Cohort | G/G | G/T+T/T | Total | OR (95% CI)^a^ | p-value | Heterogeneity |
|  |  | n (%)^c^ | n (%)^c^ | n (%)^c^ |  |  | I^2^%; p-value |
| Balance >5s-One legged | NSHD | 1742 (96.1) | 553 (96.2) | 2295 (96.1) | 1.01 (0.62- 1.65) | 0.96 |  |
| Balance >5s-Tandem | ELSA | 3719 (87.2) | 1138 (86.5) | 4857 (87.1) | 0.96 (0.78- 1.17) | 0.68 |  |
| Balance >5s-Flamingo | HCS | 966 (81.4) | 308 (83.9) | 1274 (82.0) | 1.21 (0.88- 1.66) | 0.24 |  |
| Balance >5s-Flamingo | Boyd Orr | 176 (63.5) | 57 (53.8) | 233 (60.8) | 0.65 (0.41- 1.04) | 0.07 |  |
| Balance >5s-Flamingo | CaPS | 383 (63.8) | 92 (59.0) | 475 (62.8) | 0.87 (0.60- 1.26) | 0.45 |  |
|  | **Pooled^d^** | **[6986/8141]** | **[2148/2519]** | **[9134/10660]** | **0.954 (0.809- 1.126)** | **0.58** | **19; 0.29** |

a: Beta coefficients or odds ratios based on z-scores for (G/T+T/T) vs. G/G adjusted for age and sex.

b: Reciprocal of time taken in sec x 100.

c: #participants able to balance for at least 5s (%).

d: Pooled: [#participants able to balance for at least 5s /total # participants with relevant data].

Balance, timed walk and chair rises in HCS from both phases.

**Table S6 Physical Capability by rs2941740 (*ESR1*)** Genotype and Cohort

| Variable | Cohort | A/A | A/G | G/G | Total | b (95% CI) ^a^ | p-value | Heterogeneity |
| --- | --- | --- | --- | --- | --- | --- | --- | --- |
|  |  | mean (sd) [n] | mean (sd) [n] | mean (sd) [n] | mean (sd) [n] |  |  | I^2^%; p-value |
|  | NSHD | 38.5 (14.5) [798] | 37.7 (14.2) [1215] | 37.7 (14.4) [435] | 37.9 (14.3) [2448] | -0.05 (-0.09- -0.01) | 0.0239 |  |
|  | ELSA | 31.9 (11.8) [1849] | 32.2 (11.6) [2677] | 31.9 (11.0) [989] | 32.0 (11.6) [5515] | -0.01 (-0.03- 0.02) | 0.52 |  |
| Grip strength, kg | HCS | 36.0 (11.0) [915] | 35.9 (11.0) [1376] | 35.0 (11.2) [557] | 35.7 (11.1) [2848] | -0.02 (-0.05- 0.01) | 0.20 |  |
|  | LBC1921 | 25.5 (9.2) [145] | 26.3 (9.1) [242] | 27.9 (9.0) [126] | 26.5 (9.1) [513] | 0.09 (0.01- 0.17) | 0.0219 |  |
|  | **Pooled** | **[3707]** | **[5510]** | **[2107]** | **[11324]** | **-0.008 (-0.043- 0.026)** | **0.63** | **70; 0.0180** |
| Timed 2.44m walk, m/s | ELSA | 0.92 (0.30) [1180] | 0.94 (0.29) [1691] | 0.93 (0.30) [652] | 0.93 (0.30) [3523] | 0.03 (-0.02- 0.07) | 0.21 |  |
| Timed 3m get up and go, m/s | HCS | 0.58 (0.10) [678] | 0.58 (0.09) [1024] | 0.57 (0.10) [426] | 0.57 (0.10) [2128] | -0.04 (-0.10- 0.02) | 0.19 |  |
| Timed 3m get up and go, m/s | Boyd Orr | 0.67 (0.15) [146] | 0.66 (0.17) [172] | 0.69 (0.14) [56] | 0.67 (0.16) [374] | 0.04 (-0.11- 0.18) | 0.62 |  |
| Timed 3m get up and go, m/s | CaPS | 0.61 (0.13) [260] | 0.59 (0.13) [384] | 0.60 (0.13) [108] | 0.60 (0.13) [752] | -0.07 (-0.17- 0.04) | 0.20 |  |
| Timed 6m walk, m/s | LBC1921 | 1.40 (0.39) [144] | 1.40 (0.38) [242] | 1.42 (0.34) [124] | 1.40 (0.37) [510] | 0.01 (-0.10- 0.13) | 0.81 |  |
|  | **Pooled** | **[2408]** | **[3513]** | **[1366]** | **[7287]** | **-0.004 (-0.044- 0.035)** | **0.83** | **24; 0.26** |
| Timed chair rises^b^-10 rises | NSHD | 5.18 (1.61) [779] | 5.15 (1.70) [1168] | 5.30 (1.70) [421] | 5.19 (1.67) [2368] | 0.03 (-0.03- 0.08) | 0.39 |  |
| Timed chair rises^b^-5 rises | ELSA | 9.61 (3.11) [1579] | 9.62 (3.18) [2343] | 9.69 (3.21) [875] | 9.63 (3.16) [4797] | 0.02 (-0.02- 0.06) | 0.33 |  |
| Timed chair rises^b^-5 rises | HCS | 6.22 (1.48) [467] | 6.23 (1.48) [728] | 6.32 (1.48) [316] | 6.24 (1.48) [1511] | 0.03 (-0.04- 0.10) | 0.42 |  |
|  | **Pooled** | **[2825]** | **[4239]** | **[1612]** | **[8676]** | **0.022 (-0.007- 0.050)** | **0.13** | **0.0; 0.96** |
|  |  |  |  |  |  |  |  |  |
| Variable | Cohort | A/A | A/G | G/G | Total | OR (95% CI) ^a^ | p-value | Heterogeneity |
|  |  | n (%)^c^ | n (%)^c^ | n (%)^c^ | n (%)^c^ |  |  | I^2^%; p-value |
| Balance >5s-One legged | NSHD | 773 (95.8) | 1181 (96.6) | 412 (94.5) | 2366 (96.0) | 0.89 (0.67- 1.19) | 0.44 |  |
| Balance >5s-Tandem | ELSA | 1616 (86.6) | 2350 (87.2) | 878 (87.3) | 4844 (87.0) | 1.03 (0.91- 1.16) | 0.68 |  |
| Balance >5s-Flamingo | HCS | 400 (81.8) | 613 (81.8) | 272 (82.7) | 1285 (82.0) | 1.01 (0.85- 1.22) | 0.88 |  |
| Balance >5s-Flamingo | Boyd Orr | 88 (60.7) | 106 (61.6) | 35 (62.5) | 229 (61.4) | 1.04 (0.76- 1.42) | 0.82 |  |
| Balance >5s-Flamingo | CaPS | 154 (59.2) | 257 (66.1) | 64 (57.7) | 475 (62.5) | 1.07 (0.85- 1.34)† | 0.56 |  |
|  | **Pooled^d^** | **[3031/3567]** | **[4507/5228]** | **[1661/1938]** | **[9199/10733]** | **1.018 (0.935- 1.108)** | **0.68** | **0.0; 0.91** |

†Full genotype model representing a significantly better fit than the given per allele model.

a: Beta coefficients or odds ratios based on z-scores per minor allele adjusted for age and sex.

b: Reciprocal of time taken in sec x 100.

c: #participants able to balance for at least 5s (%).

d: Pooled: [#participants able to balance for at least 5s /total # participants with relevant data].

Balance, timed walk and chair rises in HCS from both phases.

**Table S7 Physical Capability by rs9594759 (*RANKL*)** Genotype and Cohort

| Variable | Cohort | T/T | C/T | C/C | Total | b (95% CI) ^a^ | p-value | Heterogeneity |
| --- | --- | --- | --- | --- | --- | --- | --- | --- |
|  |  | mean (sd) [n] | mean (sd) [n] | mean (sd) [n] | mean (sd) [n] |  |  | I^2^%; p-value |
|  | NSHD | 37.6 (14.2) [719] | 38.1 (14.6) [1277] | 38.2 (14.0) [452] | 37.9 (14.3) [2448] | -0.02 (-0.06- 0.02) | 0.30 |  |
|  | ELSA | 32.0 (11.6) [1648] | 32.1 (11.4) [2674] | 31.9 (11.8) [1069] | 32.1 (11.6) [5391] | -0.02 (-0.04- 0.01) | 0.17 |  |
| Grip strength, kg | HCS | 35.4 (11.0) [844] | 36.1 (11.2) [1418] | 35.6 (10.8) [555] | 35.8 (11.1) [2817] | -0.00 (-0.04- 0.03) | 0.84 |  |
|  | LBC1921 | 25.8 (8.6) [160] | 27.1 (9.6) [246] | 26.5 (8.7) [93] | 26.6 (9.2) [499] | -0.00 (-0.09- 0.08) | 0.94 |  |
|  | **Pooled** | **[3371]** | **[5615]** | **[2169]** | **[11155]** | **-0.013 (-0.029- 0.004)** | **0.12** | **0.0; 0.88** |
| Timed 2.44m walk, m/s | ELSA | 0.94 (0.30) [1061] | 0.93 (0.30) [1694] | 0.92 (0.30) [697] | 0.93 (0.30) [3452] | -0.03 (-0.07- 0.01) | 0.15 |  |
| Timed 3m get up and go, m/s | HCS | 0.57 (0.09) [628] | 0.58 (0.09) [1055] | 0.57 (0.10) [411] | 0.57 (0.09) [2094] | 0.01 (-0.05- 0.07) | 0.80 |  |
| Timed 3m get up and go, m/s | Boyd Orr | 0.67 (0.15) [124] | 0.67 (0.16) [174] | 0.67 (0.15) [76] | 0.67 (0.16) [374] | -0.03 (-0.16- 0.11) | 0.70 |  |
| Timed 3m get up and go, m/s | CaPS | 0.60 (0.14) [237] | 0.60 (0.13) [347] | 0.60 (0.13) [167] | 0.60 (0.13) [751] | -0.02 (-0.12- 0.07) | 0.63 |  |
| Timed 6m walk, m/s | LBC1921 | 1.44 (0.41) [158] | 1.38 (0.36) [246] | 1.45 (0.36) [92] | 1.41 (0.38) [496] | -0.02 (-0.15- 0.10)† | 0.71 |  |
|  | **Pooled** | **[2208]** | **[3516]** | **[1443]** | **[7167]** | **-0.020 (-0.050- 0.011)** | **0.21** | **0.0; 0.90** |
| Timed chair rises^b^-10 rises | NSHD | 5.17 (1.68) [697] | 5.23 (1.70) [1241] | 5.09 (1.58) [430] | 5.19 (1.67) [2368] | -0.02 (-0.08- 0.04) | 0.49 |  |
| Timed chair rises^b^-5 rises | ELSA | 9.67 (3.15) [1454] | 9.66 (3.20) [2327] | 9.50 (3.13) [917] | 9.63 (3.17) [4698] | -0.03 (-0.06- 0.01) | 0.16 |  |
| Timed chair rises^b^-5 rises | HCS | 6.27 (1.43) [439] | 6.26 (1.51) [755] | 6.14 (1.45) [296] | 6.24 (1.47) [1490] | -0.06 (-0.13- 0.01) | 0.11 |  |
|  | **Pooled** | **[2590]** | **[4323]** | **[1643]** | **[8556]** | **-0.030 (-0.059- -0.002)** | **0.0379** | **0.0; 0.72** |
|  |  |  |  |  |  |  |  |  |
| Variable | Cohort | T/T | C/T | C/C | Total | OR (95% CI) ^a^ | p-value | Heterogeneity |
|  |  | n (%)^c^ | n (%)^c^ | n (%)^c^ | n (%)^c^ |  |  | I^2^%; p-value |
| Balance >5s-One legged | NSHD | 697 (96.0) | 1233 (95.9) | 436 (96.2) | 2366 (96.0) | 1.01 (0.75- 1.36) | 0.94 |  |
| Balance >5s-Tandem | ELSA | 1456 (87.3) | 2365 (87.7) | 920 (85.5) | 4741 (87.2) | 0.92 (0.81- 1.04) | 0.19 |  |
| Balance >5s-Flamingo | HCS | 378 (83.3) | 640 (81.6) | 249 (81.6) | 1267 (82.1) | 0.92 (0.76- 1.11) | 0.40 |  |
| Balance >5s-Flamingo | Boyd Orr | 74 (59.7) | 109 (62.6) | 45 (59.2) | 228 (61.0) | 0.93 (0.69- 1.26) | 0.64 |  |
| Balance >5s-Flamingo | CaPS | 157 (64.1) | 225 (64.8) | 96 (57.1) | 478 (62.9) | 0.84 (0.69- 1.04) | 0.11 |  |
|  | **Pooled^d^** | **[2762/3217]** | **[4572/5287]** | **[1746/2078]** | **[9080/10582]** | **0.916 (0.841- 0.997)** | **0.0417** | **0.0; 0.90** |

†Full genotype model representing a significantly better fit than the given per allele model.

a: Beta coefficients or odds ratios based on z-scores per minor allele adjusted for age and sex.

b: Reciprocal of time taken in sec x 100.

c: #participants able to balance for at least 5s (%).

d: Pooled: [#participants able to balance for at least 5s /total # participants with relevant data].

Balance, timed walk and chair rises in HCS from both phases.

**Table S8 Physical Capability by rs3815148 (*COG5*)** Genotype and Cohort

| Variable | Cohort | A/A | A/C+C/C | Total | b (95% CI)^a^ | p-value | Heterogeneity |
| --- | --- | --- | --- | --- | --- | --- | --- |
|  |  | mean (sd) [n] | mean (sd) [n] | mean (sd) [n] |  |  | I^2^%; p-value |
|  | NSHD | 38.2 (14.5) [1427] | 37.6 (14.1) [1021] | 37.9 (14.3) [2448] | -0.08 (-0.14- -0.02) | 0.0082 |  |
|  | ELSA | 32.0 (11.5) [3299] | 32.2 (11.7) [2221] | 32.0 (11.6) [5520] | 0.01 (-0.02- 0.04) | 0.60 |  |
| Grip strength, kg | HCS | 35.6 (11.1) [1668] | 36.0 (10.9) [1172] | 35.8 (11.0) [2840] | -0.02 (-0.07- 0.02) | 0.34 |  |
|  | LBC1921 | 26.5 (9.1) [292] | 26.5 (9.2) [221] | 26.5 (9.1) [513] | -0.01 (-0.13- 0.10) | 0.80 |  |
|  | **Pooled** | **[6686]** | **[4635]** | **[11321]** | **-0.023 (-0.063- 0.016)** | **0.25** | **56; 0.08** |
| Timed 2.44m walk, m/s | ELSA | 0.93 (0.30) [2090] | 0.93 (0.30) [1433] | 0.93 (0.30) [3523] | -0.00 (-0.06- 0.06) | 0.98 |  |
| Timed 3m get up and go, m/s | HCS | 0.57 (0.09) [1230] | 0.57 (0.10) [890] | 0.57 (0.09) [2120] | -0.01 (-0.10- 0.07) | 0.75 |  |
| Timed 3m get up and go, m/s | Boyd Orr | 0.67 (0.15) [249] | 0.66 (0.16) [134] | 0.67 (0.16) [383] | -0.06 (-0.27- 0.14) | 0.54 |  |
| Timed 3m get up and go, m/s | CaPS | 0.59 (0.13) [455] | 0.61 (0.13) [296] | 0.60 (0.13) [751] | 0.10 (-0.04- 0.24) | 0.17 |  |
| Timed 6m walk, m/s | LBC1921 | 1.38 (0.37) [290] | 1.44 (0.38) [220] | 1.40 (0.37) [510] | 0.15 (-0.02- 0.32) | 0.08 |  |
|  | **Pooled** | **[4314]** | **[2973]** | **[7287]** | **0.019 (-0.037- 0.074)** | **0.50** | **23; 0.27** |
| Timed chair rises^b^-10 rises | NSHD | 5.20 (1.63) [1389] | 5.17 (1.73) [979] | 5.19 (1.67) [2368] | -0.03 (-0.11- 0.06) | 0.55 |  |
| Timed chair rises^b^-5 rises | ELSA | 9.61 (3.16) [2862] | 9.67 (3.18) [1939] | 9.64 (3.17) [4801] | 0.02 (-0.03- 0.07) | 0.46 |  |
| Timed chair rises^b^-5 rises | HCS | 6.20 (1.48) [882] | 6.31 (1.48) [622] | 6.25 (1.48) [1504] | 0.05 (-0.05- 0.15) | 0.31 |  |
|  | **Pooled** | **[5133]** | **[3540]** | **[8673]** | **0.014 (-0.026- 0.054)** | **0.50** | **0.0; 0.48** |
|  |  |  |  |  |  |  |  |
| Variable | Cohort | A/A | A/C+C/C | Total | OR (95% CI)^a^ | p-value | Heterogeneity |
|  |  | mean (sd) [n] | mean (sd) [n] | mean (sd) [n] |  |  | I^2^%; p-value |
| Balance >5s-One legged | NSHD | 1387 (96.0) | 979 (96.0) | 2366 (96.0) | 0.99 (0.66- 1.49) | 0.95 |  |
| Balance >5s-Tandem | ELSA | 2883 (86.7) | 1967 (87.6) | 4850 (87.1) | 1.08 (0.90- 1.29) | 0.40 |  |
| Balance >5s-Flamingo | HCS | 744 (82.2) | 537 (82.1) | 1281 (82.2) | 0.98 (0.75- 1.28) | 0.87 |  |
| Balance >5s-Flamingo | Boyd Orr | 153 (61.7) | 79 (59.0) | 232 (60.7) | 0.89 (0.57- 1.39) | 0.61 |  |
| Balance >5s-Flamingo | CaPS | 288 (62.9) | 189 (62.8) | 477 (62.8) | 0.98 (0.71- 1.33) | 0.87 |  |
|  | **Pooled^d^** | **[5455/6382]** | **[3751/4354]** | **[9206/10736]** | **1.017 (0.901- 1.149)** | **0.78** | **0.0; 0.92** |

a: Beta coefficients or odds ratios based on z-scores for (A/C+C/C) vs. A/A adjusted for age and sex.

b: Reciprocal of time taken in sec x 100.

c: #participants able to balance for at least 5s (%).

d: Pooled: [#participants able to balance for at least 5s /total # participants with relevant data].

Balance, timed walk and chair rises in HCS from both phases.


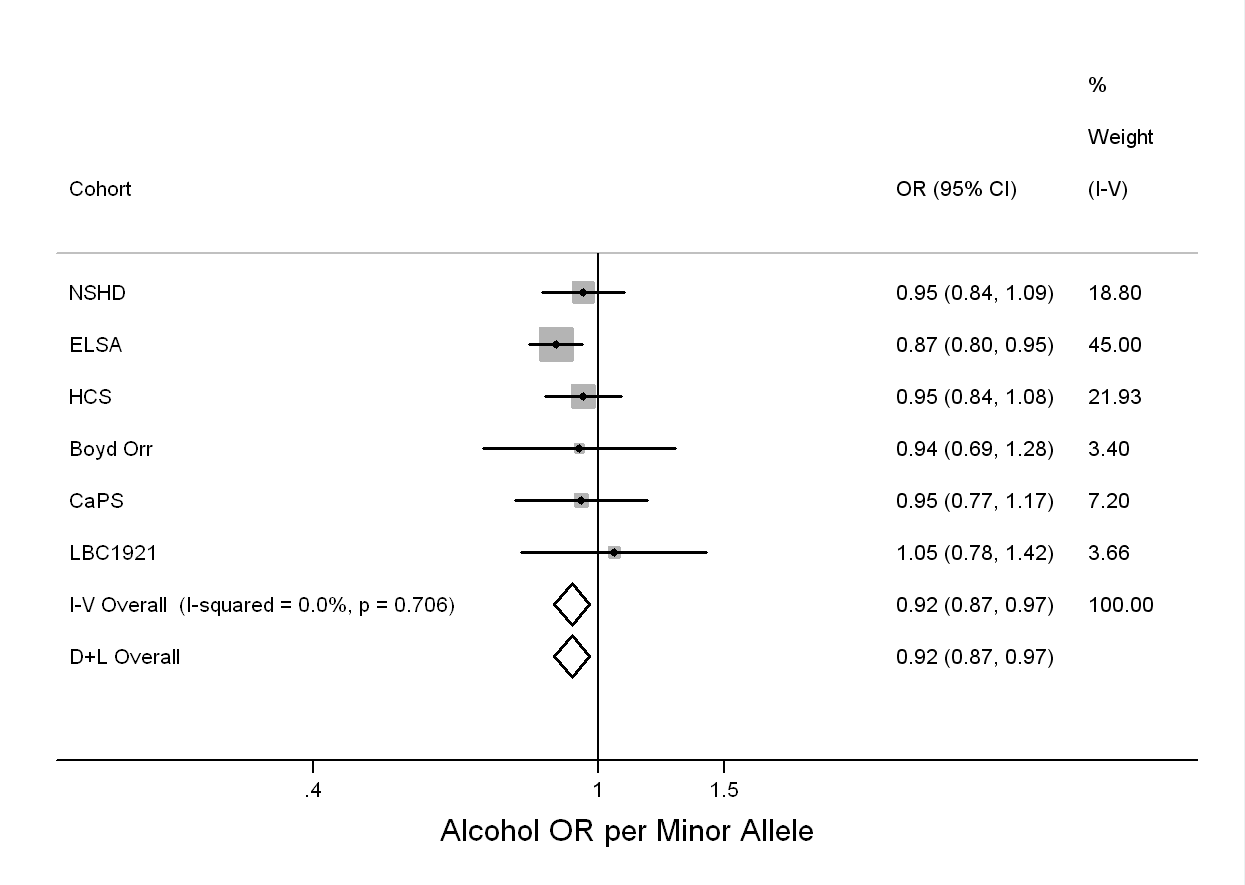


**Figure S1 Meta-analysis for the Association between rs9594759 (*RANKL*) and Alcohol Consumption**

Adjusted for age and sex. Odds ratio for at least weekly alcohol consumption, or ‘more frequently than on special occasions’ in NSHD. Odds ratio per C allele.


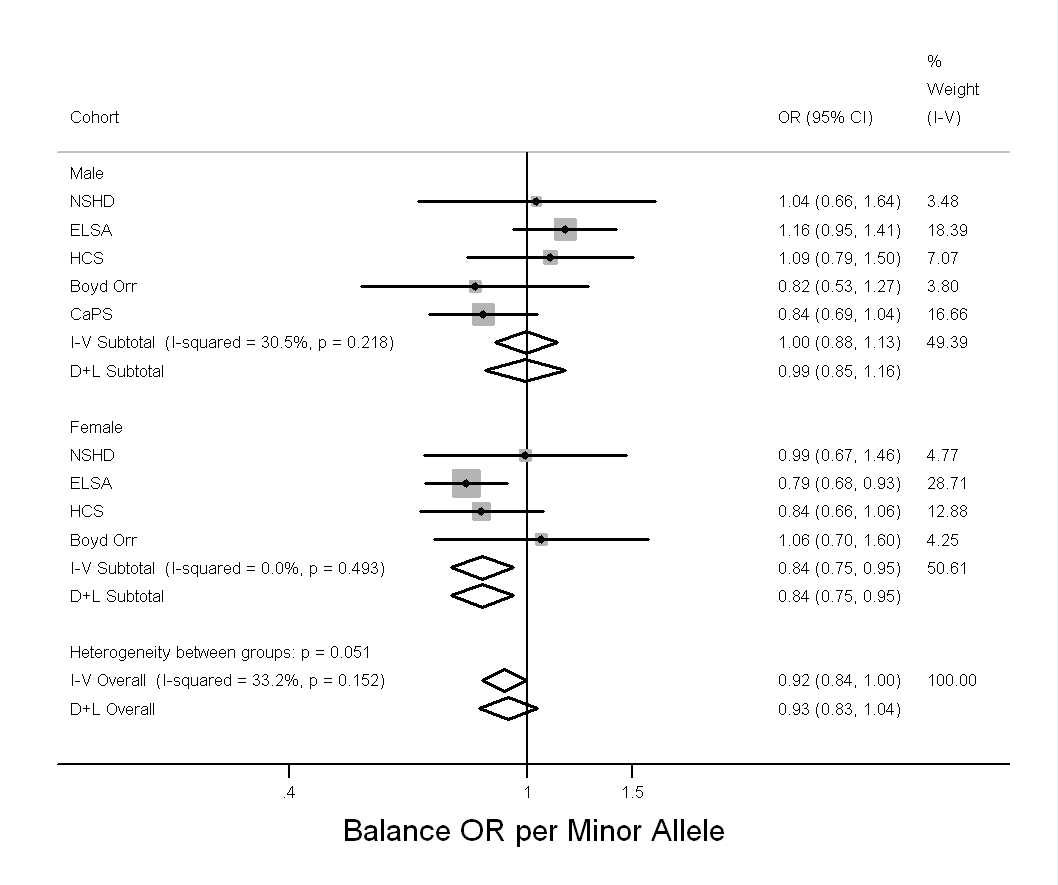


**Figure S2 Meta-analysis for the Association between rs9594759 (*RANKL*) and Ability to Balance for at Least 5s by Sex**

Adjusted for age.


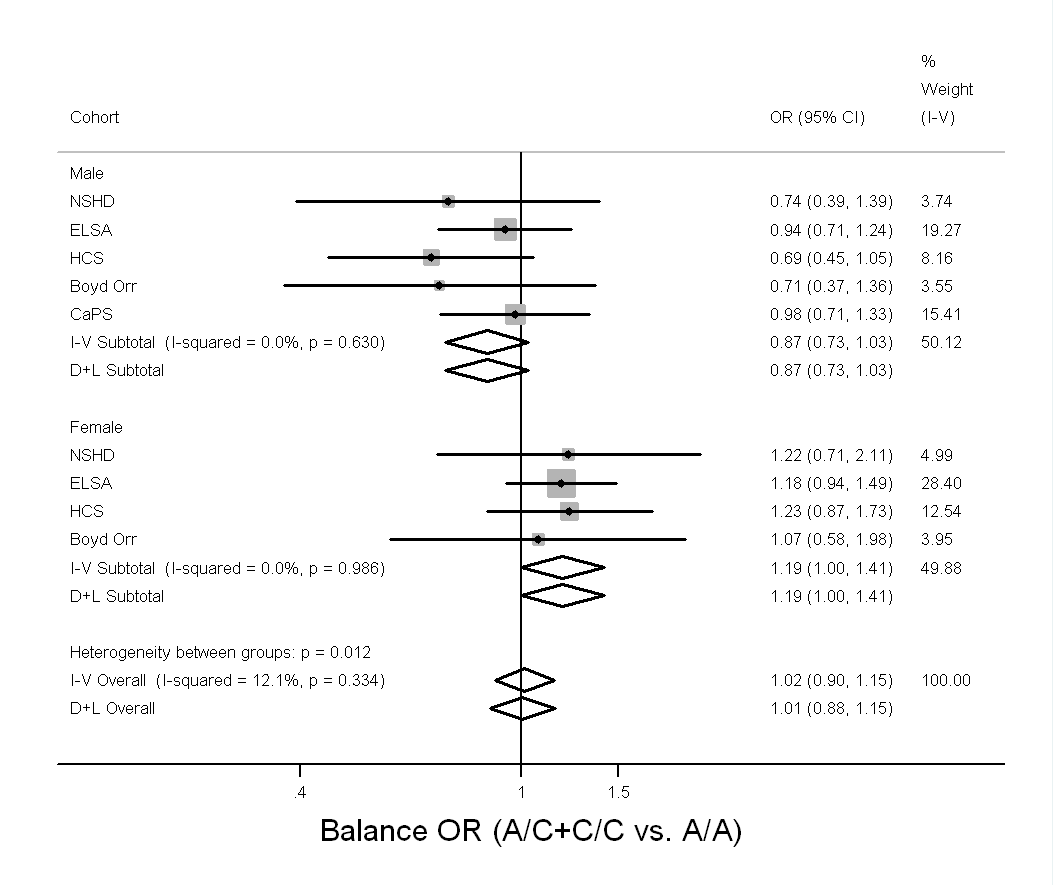


**Figure S3 Meta-analysis for the Association between rs3815148 (*COG5*) and Ability to Balance for at Least 5s by Sex**

Adjusted for age.
